# Comprehensive study on in vitro propagation of some imported peach rootstocks b. In vitro rooting and acclimatization

**DOI:** 10.1038/s41598-025-00848-z

**Published:** 2025-05-23

**Authors:** Galal I. Eliwa, El Refaey F. El Dengawy, Mohamed S. Gawish, Mona M. Yamany

**Affiliations:** Horticulture Department, Faculty of Agriculture, Damietta University, New Damietta, Egypt

**Keywords:** Acclimatization, Indole-3-butyric acid, Genotype, Micropropagation, Biotechnology, Physiology, Plant sciences

## Abstract

Rooting is considered a critical stage in the micropropagation of *Prunus sp*. because it controls plant survival during acclimatization. Auxins and genotype are thought to play a significant role in root formation and induction. Therefore, the objective of the present work was to evaluate the effect of different indole-3-butyric acid (IBA) concentrations on the in vitro rooting and acclimatization of the Okinawa, Nemared, and Garnem peach rootstocks. Microcuttings of 2–3 cm in length were cultivated in MS medium supplemented with IBA (0.0, 0.5, 1.0, 2.0, 3.0, and 4.0 mg L^− 1^) for the in vitro rooting stage. According to our results, the Garnem genotype exhibited the highest in vitro rooting rate, number of roots per plantlet, and root length. The level of 2.0 mg L^− 1^ of IBA was associated with rooting rates of 100%, 83.33%, and 75% for the Garnem, Okinawa, and Nemared, respectively. The Garnem genotype responded to 4.0 mg L^− 1^ of IBA with a fixed highest root number (12.33), which was the average number of roots per plantlet. Whereas, for the Nemared and Okinawa genotypes, the highest root number per plantlet was 8.00 and 5.00, respectively, in response to 3.0 mg L^− 1^ of IBA. The root lengths of the three genotypes varied significantly depending on the IBA treatment. The Garnem genotype presented the longest root length (5.33 cm), which was followed by the Okinawa genotype (2.49 cm), while the shortest value was presented with the Nemared (1.43 cm). The current study also demonstrated that the three genotypes developed abnormal roots and callus formation when the IBA concentration was increased to 4.00 mg L^− 1^. Following acclimatization, the Garnem, Okinawa, and Nemared genotypes had respective average survival rates of 93%, 90%, and 75% for plantlets with fully grown shoots and roots.

## Introduction

The peach (*Prunus persica L. Batsch*) is the third most important temperate tree fruit species after apples and pears^1^. Egypt is the eighth-largest producer of peaches in the world, after China and the European Union, with a total production of 244,228.55 tons^2^. However, root-knot nematodes are a major problem that can limit the expansion of peach growing in different regions of Egypt, especially in sandy soils^3^. The most common management methods for root-knot nematodes are chemical nematicides, which are expensive and pollute the environment^4^. Eco-friendly techniques, like biological control and resistant rootstocks, have therefore drawn particular interest^5^. Egypt spends millions of dollars every year importing peach rootstocks that are resistant to root-knot nematodes, like Nemaguard, Garnem, Nemared, and Okinawa, as seeds or tissue culture seedlings from the USA, Italy, Spain, and France for budded stone fruits (peaches, nectarines, plums, and almonds)^3^.

It is difficult to propagate peach rootstocks on a large scale using soft, hardwood, or greenwood cuttings due to their low rooting capacity^3,6,7^,^8^. Micropropagation offers a practical means of providing growers with sufficient quantities of pathogen-free rootstocks^9^. Micropropagation of rooting is regarded as a crucial stage in *Prunus* species since it affects the plant’s ability to survive acclimatization. Auxins and the genotype are two significant rooting-related factors that are thought to be crucial for the induction and development of roots^10,11^.The researchers confirmed that auxins can basically stimulate adventitious roots on explanted plants^12,13^. Therefore, the addition of exogenous auxins is required in the early stages to stimulate root formation^14,15^ and the emergence of numerous roots^16^. The number of roots is a significant factor for increased plant survival percentage during the acclimatization phase, and it is considered a qualitative trait of rooting response^17^.

One of the most crucial stages in the propagation of in vitro cultures is selecting the proper kind and concentration of plant growth regulator (PGR)^18,19^. For this, a variety of synthetic auxins can be employed, including naphthalene acetic acid (NAA) and indole-3-butyric acid (IBA), while the primary naturally occurring auxin in plants is indole-3-acetic acid (IAA)^18^. It has been reported that IBA is the most effective method for inducing roots in *Prunus* species^13,20^,^21^. One possible explanation for IBA’s superior performance over NAA and IAA is its delayed degradation and slow migration in the cultivation medium^12^. Additionally, it is less susceptible to enzymes that break down auxin and would be gradually broken down by the peroxidase enzyme^15,22^. Numerous researchers have encountered issues because in vitro-grown roots may exhibit physiological disorders despite having normal morphology^10,23^. Root induction in the *Prunus sp*. can be affected by an interaction of factors, highlighting the genotype^24^, the culture medium^25–30^, and growth regulators, among which auxins are decisive for successful rooting in vitro^31–34^. Additionally, some species in the *Prunus* genus are hesitant to root in vitro and show a low frequency of root formation, which presents vast economic consequences. Therefore, we must also optimize tissue culture systems for the rooting of these species.

The plants are able to adjust to ex-vitro conditions because the acclimatization is carried out gradually. Usually, the plants are taken from high to low humidity and low to high light intensity^35,36^. Successful acclimatization of the plantlets required the use of IBA to induce healthy and well-developed roots while in in vitro culture^34,37^. Micropropagation on the current rootstocks has been the subject of relatively few studies in Egypt. Additionally, the tissue culture cycle has encountered numerous issues, including explant contamination, veterinary verification, and the need to modify plant growth regulators during the proliferation, rooting stages, and acclimatization^38^. Our objective was to establish a protocol for the rapid and economical micropropagation of the Garnem, Nemared, and Okinawa peach rootstocks with the goal of lessening our dependency on imports and maintaining hard currency for our country. The present investigation examined the rooting ability in vitro and acclimatization of the Okinawa, Garnem, and Nemared *peach* rootstocks in response to varying IBA concentrations.

## Materials and methods

### Plant material and explant Preparation

This study was conducted in the tissue culture laboratory of the Horticulture Department, Faculty of Agriculture, Damietta University, Egypt, from May 2020 to June 2023 to micropropagate the following three rootstocks that are recommended to Egypt conditions as below. Experimental research on plants, including collection of plant material, was performed in accordance with the relevant guidelines and regulations.

“Okinawa” (*P. persica*) originated in Gainesville, Florida, from an imported seed from Japan, and it was taken to the United States in 1953, where it was selected as rootstock from seeds. Its chilling requirement is low (150 CU), used as a source for breeding in adaptation of a low-chill peach cultivar, resistance to *Meloidgyne incognita*,* M. javanica*, and tolerance to *M. floridensis*^39^.

‘‘Nemared” Orig. at Fresno, Calif., by J.H. Weinberger, USDA; introduced in 1983 by D.W. Ramming. An F3 derivative from Nemaguard × a red-leaved seedling of Tennessee Natural. Tested as P115-95. A very red-leaved seedling rootstock. More tolerant of *Meloidogyne incognita* and *M. javanica* than Nemaguard. In the nursery, liners produce fewer side branches and can be budded earlier than Nemaguard. Pixy. -Not compatible with peaches^40^.

“Garnem” [*Peach amygdalus Batsch*, syn. *P. dulcis* (Mill.) D.A. Webb] X [*P*. *persica (L.) Btsch*] is characterized by red leaves, good vigor, easy clonal propagation, resistance to root-knot nematodes, adaptation to calcareous soils and other Mediterranean agroecological conditions, and graft compatibility with the whole range of peach and almond cultivars as well as some plum and apricot cultivars^41^.

#### Rooting stage

In this report, we have examined the rooting capacity of Okinawa, Garnem, and Nemared *peach* rootstocks in vitro in response to varying concentrations of indole butyric acid (IBA), following studies on an explant sterilization procedure and assessment of the rootstocks’ capacity to multiply in vitro under the influence of different BAP concentrations in conjunction with IBA^38^. For in vitro rooting, 2–3 cm long microcuttings were added to a rooting medium that contained half the strength of MS^42^ plus 3% (w/v) sucrose, 3 g/L gerlite, 1.5 g/L activated charcoal, and varying amounts of indole-butyric acid (IBA) at 0.0, 0.5, 1.0, 2.0, 3.0, and 4.0 mg L^− 1^. The control was the MS medium devoid of IBA. The experiment was conducted in a growth chamber with a photoperiod of 16 h of light using cool-white fluorescent lamps (light intensity of 2000 lx), a temperature of 23 ± 2 °C, and a relative humidity (RH) of 70 to 80%. The experimental design adopted was completely randomized (CRD) in a 3 × 6 factorial scheme (three genotypes combined with six concentrations of IBA: 0.0, 0.5, 1.0, 2.0, 3.0, and 4.0 mg L^− 1^. Each experimental unit consisted of nine jars, each with one microcutting with three replications. After 6 weeks, the rooting percentage was calculated for each treatment. Also, the number of roots per plantlet and average root length (cm) were measured and recorded. The average length (cm) of the root was estimated with the aid of a caliper and recorded.

#### Acclimatization stage

**Fig. 1:**
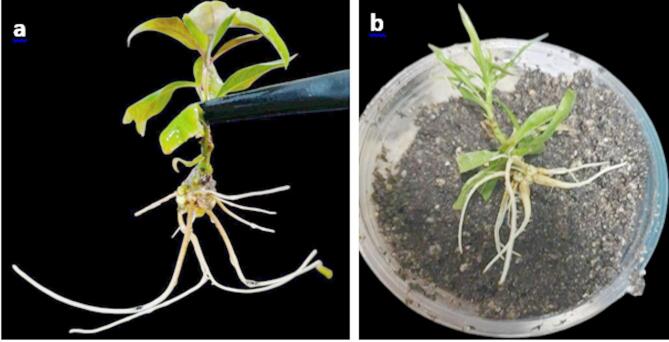
Acclimatization of plantlets with fully grown roots and shoots: (**a**) Plantlet following removal from the medium with some gerlite; (**b**) Plantlet following cleaning of the residual medium.

Plantlets with fully grown roots and shoots (Fig. 1) were carefully removed from the propagation jars and thoroughly cleaned with tap water to remove any, particularly the hardened material, gerlite, which could be the source of contamination. The roots were then placed in the fungicide Topsin M70. Then transplanted in plastic cups (5 cm in diameter) containing a soil mixture consisting of peat moss + perlite + sand (1:1:1 (v/v/v) ratio). The soil mixture was autoclaved at 1.05 kg/cm² and 121 °C for 20 min to sterilize it. These cups have four holes in the bottom to get rid of excess irrigation water; each cup contains one plantlet. The plantlets were irrigated with tap water as needed, and the cups were covered after planting (to reduce moisture loss and protect the plantlets from drying out) and placed in the growth room with a temperature of 25 ± 2 °C and a photoperiod of 16 h of light provided by cool-white fluorescent lamps (light intensity of 2000 lx). The plantlets were fertilized weekly with a quarter-strength MS solution, once a week. After 6 weeks, the survival rate was recorded as the following equation:

Number of survived plantlets

$${\text{Survival }}\,{\text{plants }}\% {\text{ }}=\frac{{{\text{Number}}\,{\text{ of }}\,{\text{survived }}\,{\text{plantlets}}}}{{{\text{Total}}\,{\text{ number}}\,{\text{ of}}\,{\text{ acclimatized }}\,{\text{plantlets}}}} \times 100$$

### Statistical analysis

In a completely randomized (CRD) design, the experiments were conducted as factorials, and the results were validated by repeating them three times. Each experimental unit consisted of nine jars, each with one microcutting with three replications. CoStat Computer Software (version 6.311) was used to analyze average data about the effects of IBA concentrations for each genotype. The least significant difference (LSD) test at *p* ≤ 0.05 was used to assess mean differences^43^.

## Results and discussion

### Rooting stage

#### The effect of IBA on average rooting percentage of the Okinawa, Garnem, and Nemared Peach rootstocks

**Fig. 2:**
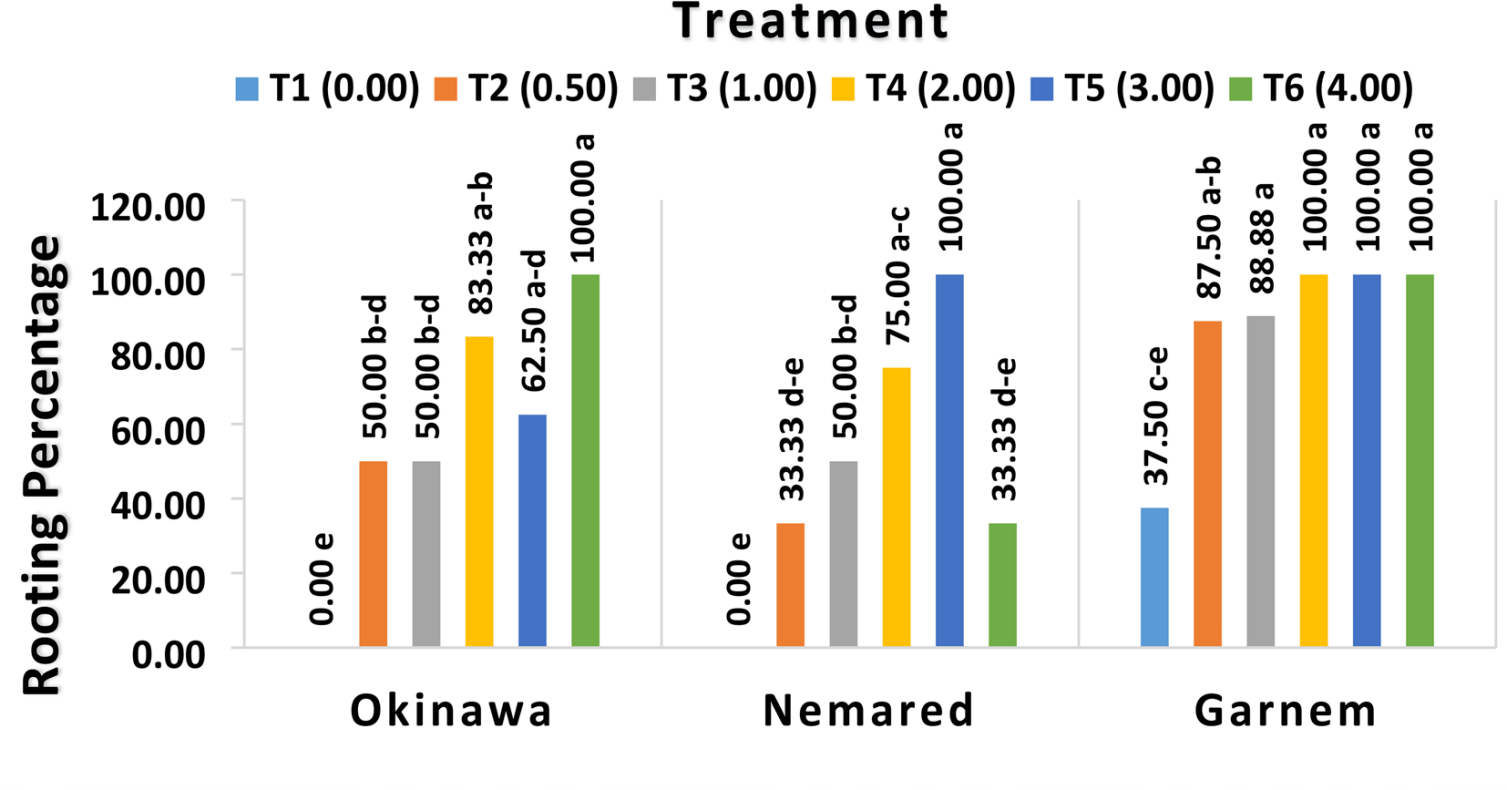
The effect of varying IBA concentrations on average rooting percentage of the Okinawa, Garnem, and Nemared peach rootstocks.

Tables 1and Figure 2 display the average rooting percentages of the Okinawa, Garnem, and Nemared peach rootstocks as influenced by varying IBA concentrations. The in vitro rooting percentages of the Okinawa, Garnem, and Nemared peach rootstocks showed distinct and noteworthy variations depending on the tested IBA concentrations. The lowest significant value of average rooting percentage (12.50%) was recorded with the control T1 (0.00 mg L^− 1^ IBA), while the high significant values of average rooting percentage (87.50% and 86.11%) were recorded with T5 (3.00 mg L^− 1^ IBA) and T4 (2.00 mg L^− 1^ IBA), respectively.

**Table 1:** The effect of varying IBA concentrations on average rooting percentage of the Okinawa, Garnem, and Nemared Peach rootstocks.

Treatment (B)	Rootstock (A)			
	Okinawa	Nemared	Garnem	Average (B)
T1 (0.00 mg L^− 1^ IBA)	0.00 e	0.00 e	37.50 c–e	12.50 C
T2 (0.5 mg L^− 1^ IBA)	50.00 b–d	33.33 de	87.50 ab	56.94 B
T3 (1.00 mg L^− 1^ IBA)	50.00 b-d	50.00 b-d	88.88 a	62.96 B
T4 (2.00 mg L^− 1^ IBA)	83.33 ab	75.00 a-c	100.00 a	86.11 A
T5 (3.00 mg L^− 1^ IBA)	62.50 a–d	100.00 a	100.00 a	87.50 A
T6 (4.00 mg L^− 1^ IBA)	100.00 a	33.33 de	100.00 a	77.77 AB
Average (A)	57.63 B	48.61 B	85.64 A	

*The LSD test shows that the means of each factor and their interaction, denoted by the same letters, are not significantly different from one another at *P* ≤ 0.05.

Regarding the behavior of genotype rootstock as influenced by IBA concentration on average rooting percentage, the data shown in Table 1; Fig. 2 made it evident that genotype has a significant impact on rooting percentage. As the average rooting percentage was the highest for the Garnem genotype (85.64%) and the lowest for the Nemared genotype (48.61%), there was no discernible difference with the Okinawa genotype (57.63%), suggesting that the rooting percentage is genotype-dependent. These results are in line with those of^44^, who discovered that the Gisela 5 genotype had the lowest value and that Fereley Jaspi had the highest rooting ability (100%) among genotypes, followed by Pyro dwarf and Gisela 6 (both of which had the best rooting percentage of 90%). while the Gisela 5 genotype had the lowest value (70%) among genotypes. Also^45^, showed the highest rooting rate (60 and 75%, respectively) of the GF677 hybrid rootstock and Rabi cultivar.

With respect to the genotype-IBA concentration interaction, the data presented in Table 1; Fig. 2 demonstrated that the genotype-IBA concentration interaction strongly influenced rooting percentage. Depending on the tested IBA concentrations, the rootstocks responded differently to the rooting percentage. In terms of the rate of rooting (100%) at concentrations T4 (2.00 mg L^− 1^ IBA), T5 (3.00 mg L^− 1^ IBA), and T6 (4.00 mg L^− 1^ IBA), respectively, the Garnem genotype recorded the highest significant values. The Nemared genotype recorded the same rate (100%) in T5 (3.00 mg L^− 1^ IBA), while the Okinawa genotype recorded the average rooting (100%) with the highest concentration of IBA in T6 (4.00 mg/L^− 1^ IBA). The lowest value of rooting percentage (0.00%) was recorded with the Okinawa and Nemared genotypes in the control treatment T1 (0.00 mg L^− 1^ IBA), while the Garnem genotype recorded 37.50% of the rooting rate in the control treatment (T1 without IBA), and that could be attributed to the Garnem genotype having enough endogenous cytokines and auxin combination for root initiation^46^.

#### The effect of varying IBA concentrations on average root length (cm)

**Fig. 3:**
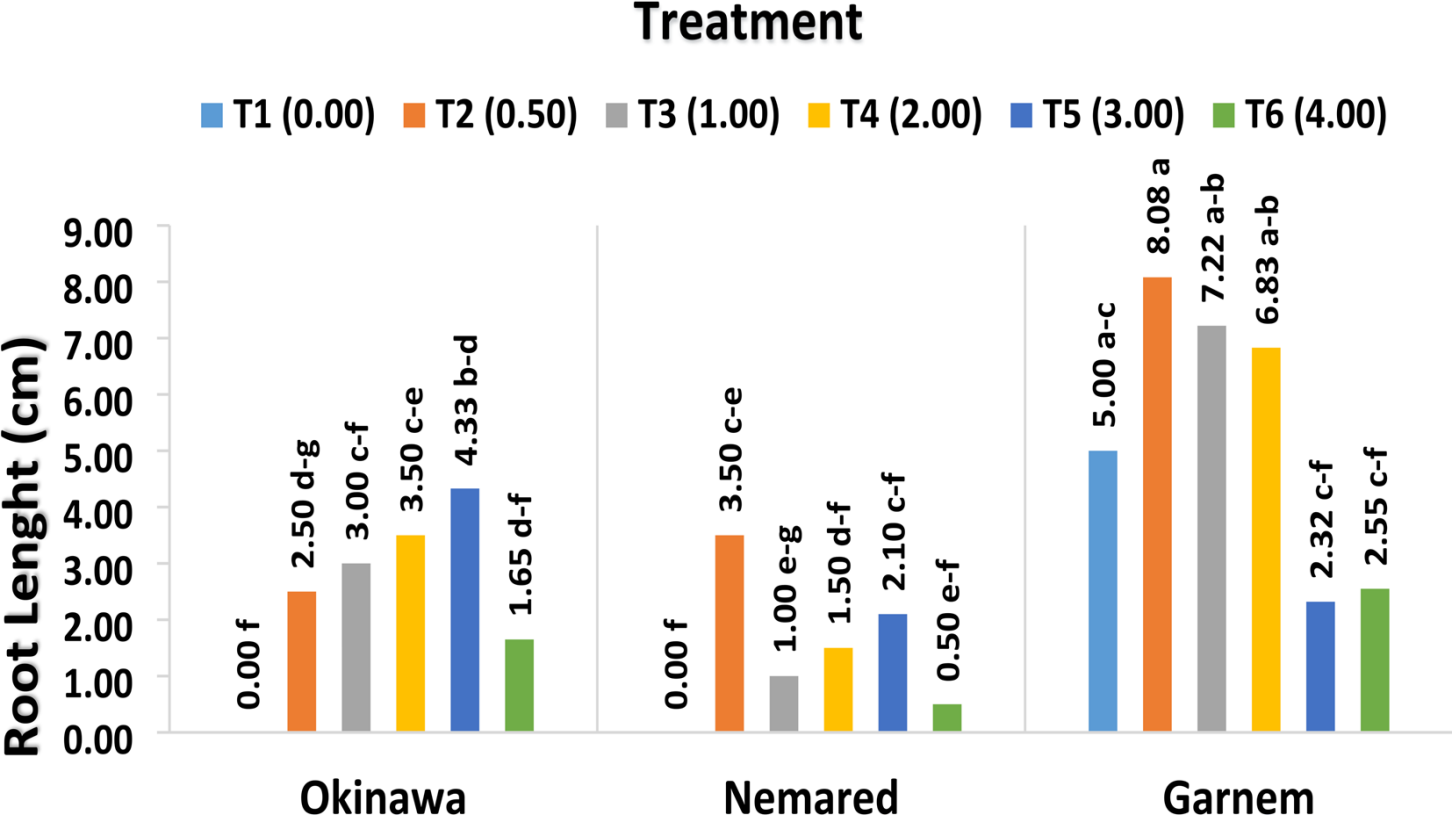
The effect of varying IBA concentrations on average root length (cm) of the Okinawa, Garnem, and Nemared peach rootstocks.

The effect of IBA concentrations on the average root length (cm) of the Okinawa, Garnem, and Nemared *peach* rootstocks was evaluated in Table 2; Fig. 3. According to the results, explants grown on medium T2 (0.05 mg L^− 1^ IBA) had the longest average root length (4.69 cm), while those grown on medium T6 (4.00 mg L^− 1^ IBA) had the shortest average root length (1.56 cm).

**Fig. 4:**
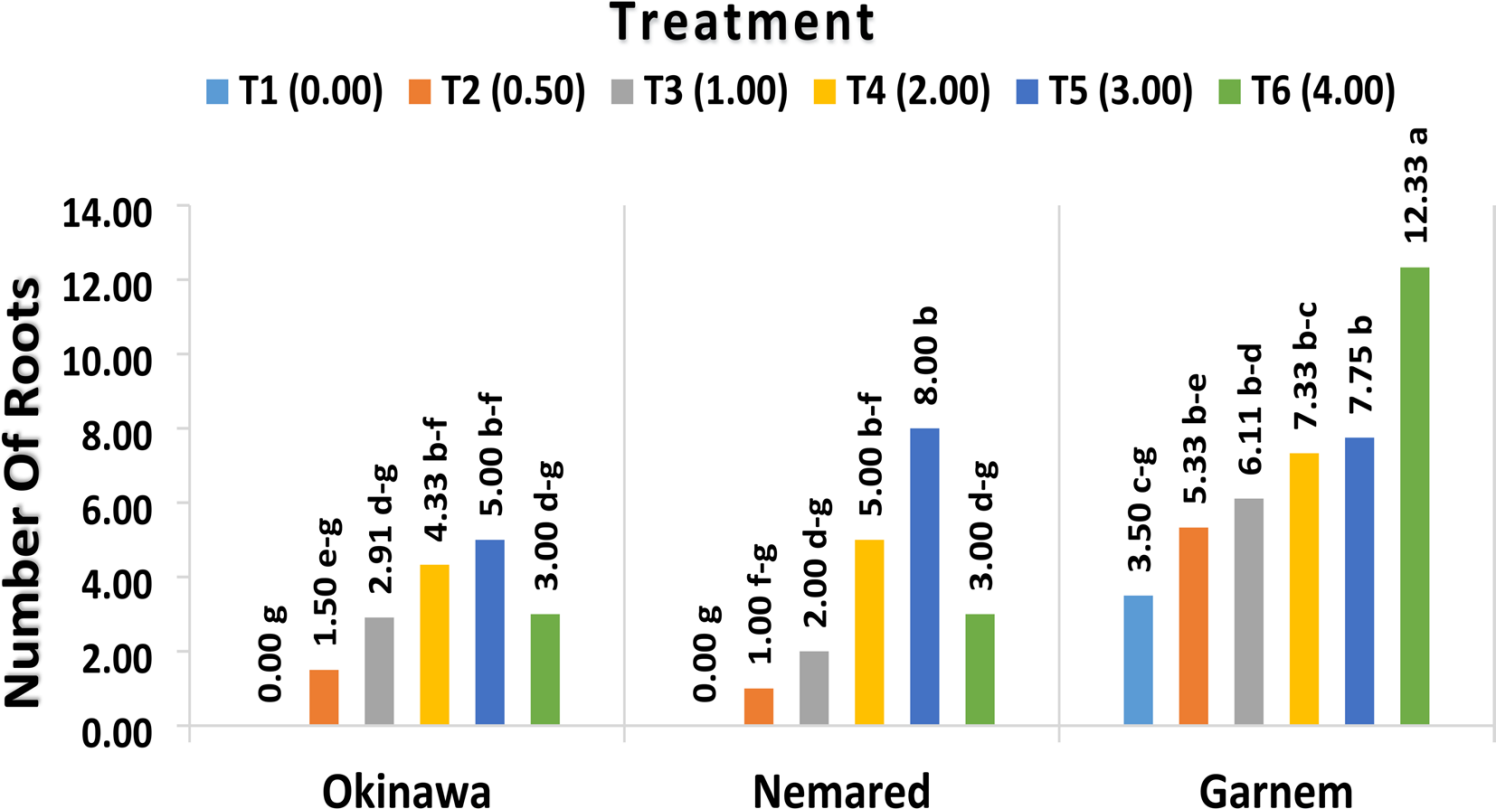
The effect of varying IBA concentrations on the average number of roots per plantlet of the Okinawa, Nemared, and Garnem peach rootstocks.

**Fig. 5:**
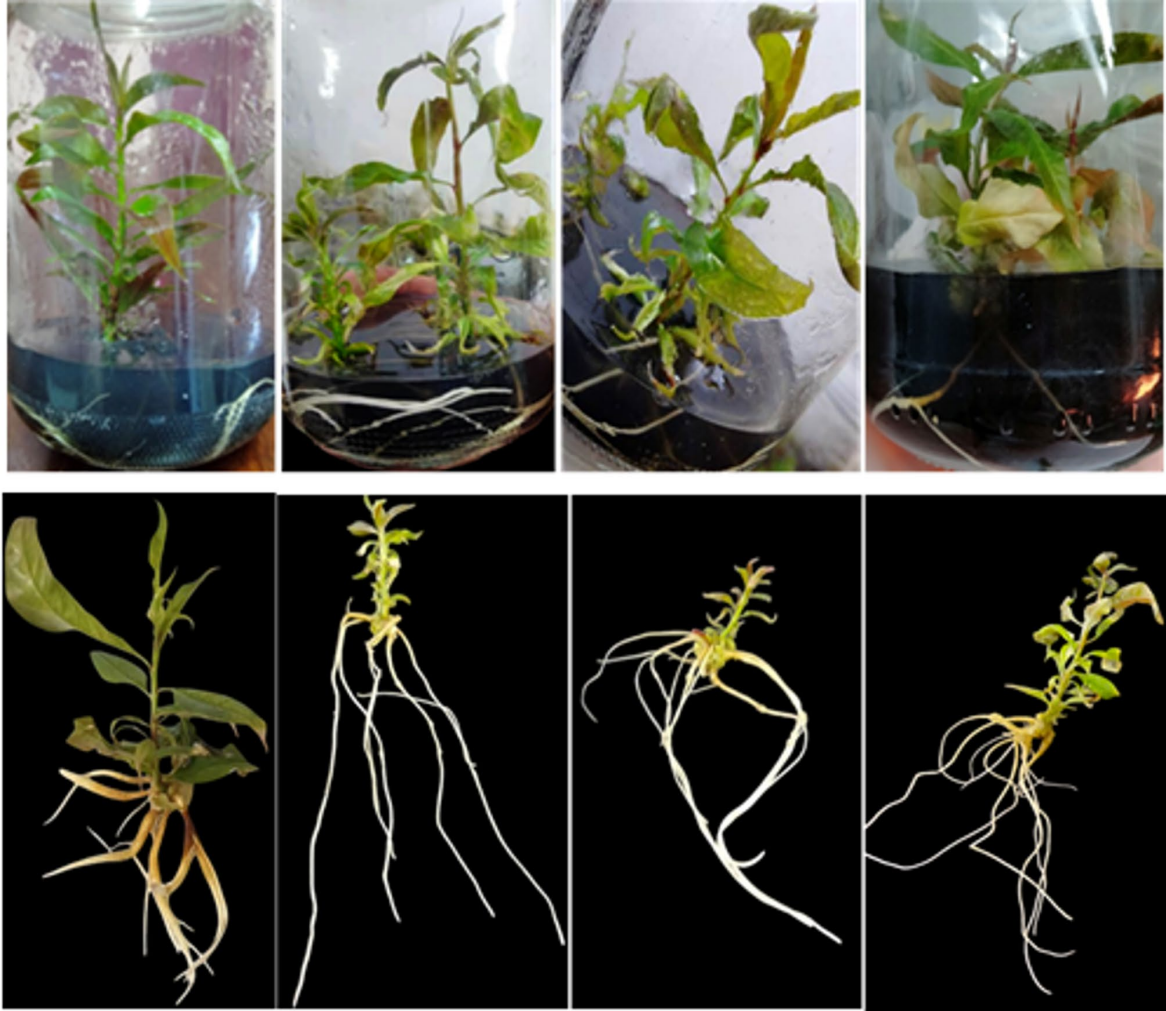
Effect of 2.0 mg L^− 1^ of IBA on the average number of roots per plantlet of Garnem peach rootstock.

**Fig. 6:**
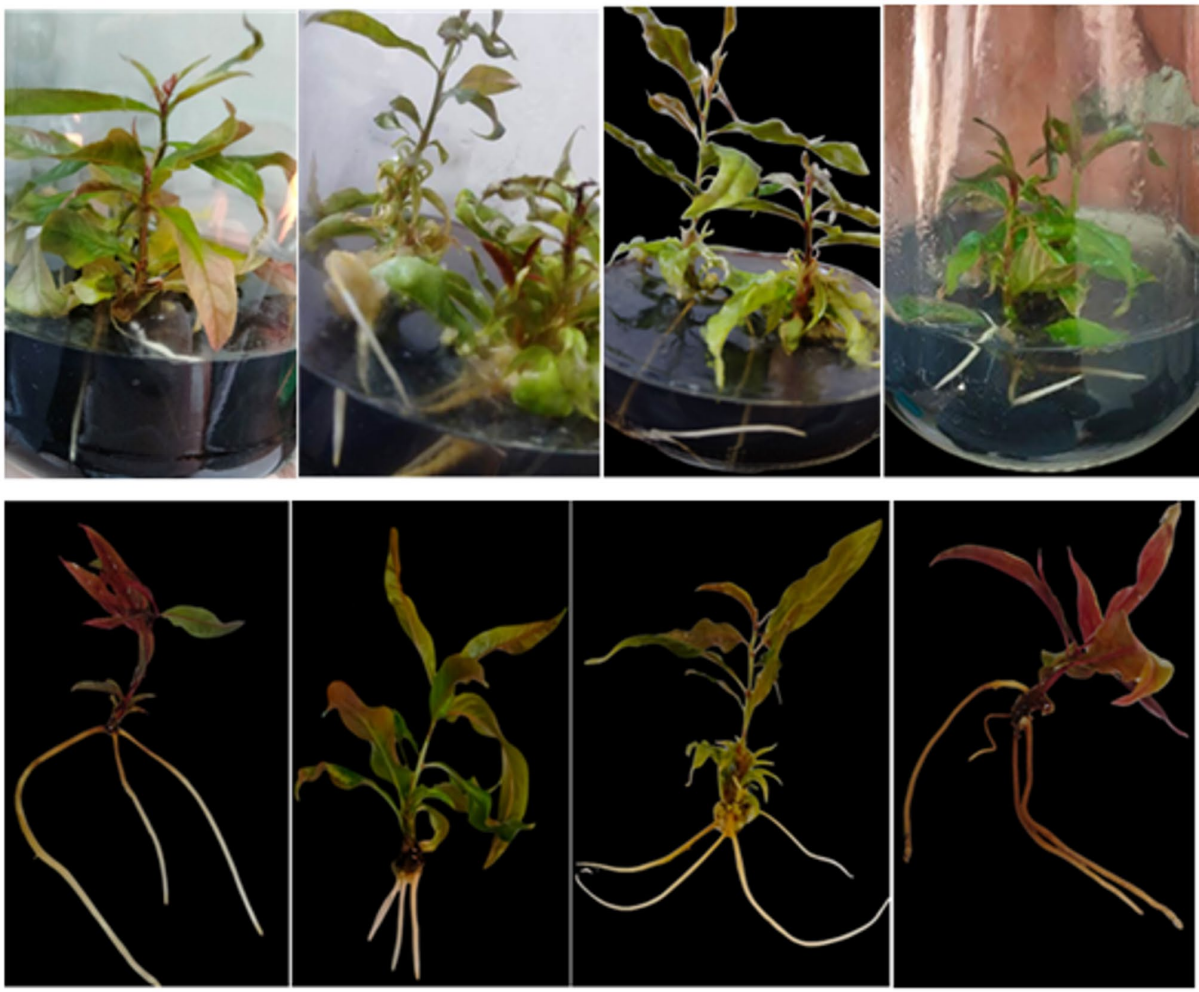
The effect of 2.0 mg L^− 1^ of IBA on the average number of roots per plantlet of Nemared peach rootstock.

Concerning the genotype rootstock behavior as influenced by IBA on average root length (cm), the data shown in Table 2; Figs. 3, 4, 5 and 6 made it evident that the three rootstocks had a significant difference in root length as influenced by the IBA treatments. The Garnem genotype produced the longest root length (5.33 cm), which was significantly followed by the Okinawa genotype (2.49 cm), while the shortest average root length (1.43 cm) was recorded with the Nemared genotype.

**Fig. 7:**
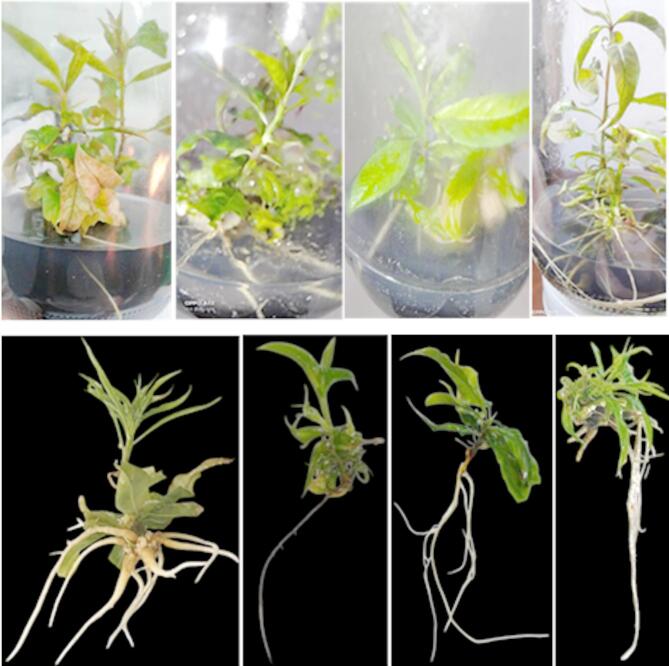
The effect of 2.0 mg L^− 1^ of IBA on the average number of roots per plantlet of Okinawa peach rootstock.

Regarding the interaction between IBA concentration and genotypes on average root length per plantlet, the results in Table 2; Figs. 3, 4, 5 and 6 cleared that the rootstocks showed different responses to the average root length (cm) with relation to the IBA levels. The Garnem genotype recorded the highest significant root length (8.08 cm) with T2 (0.5 mg L^− 1^ IBA), followed by the Okinawa genotype, which recorded root length (4.33 cm) in T5 (3.00 mg L^− 1^ IBA).

**Table 2:** The effect of varying IBA concentrations on average root length (cm) of the Okinawa, Garnem, and Nemared *Peach* rootstocks.

Treatment (B)	Rootstock (A)			
	Okinawa	Nemared	Garnem	Average (B)
T1 (0.00 mg L^− 1^ IBA)	0.00 f	0.00 f	5.00 a–c	1.66 B
T2 (0.5 mg L^− 1^ IBA)	2.50 d–g	3.50 c–e	8.08 a	4.69 A
T3 (1.00 mg L^− 1^ IBA)	3.00 c–f	1.00 e–g	7.22 ab	3.74 A
T4 (2.00 mg L^− 1^ IBA)	3.50 c–e	1.50 d–f	6.83 ab	3.94 A
T5 (3.00 mg L^− 1^ IBA)	4.33 b–d	2.10 c–f	2.32 c–f	2.92 AB
T6 (4.00 mg L^− 1^ IBA)	1.65 d–f	0.50 e–f	2.55 c–f	1.56 B
Average (A)	2.49 B	1.43 B	5.33 A	

*The LSD test shows that the means of each factor and their interaction, denoted by the same letters, are not significantly different from one another at *P* ≤ 0.05.

Our results are in agreement with^40^, who showed that the maximum root length in the medium containing 0.5 mg L^− 1^ IBA of GF677. The longest roots were found in *P. salicina* media supplemented with 1.0 mg L-1 IBA, according to^48^.

#### The effect of varying IBA concentrations on average number of roots per plantlet

The effect of IBA treatments on the average root number per plantlet of the Okinawa, Garnem, and Nemared peach rootstocks was cleared in Table 3; Fig. 7. The IBA had a positive effect on the in vitro rooting of the evaluated rootstocks, significantly affecting the number of roots per plantlet. The high significant values (6.91 and 6.11) of the average root number were recorded with high concentrations of IBA T5 (3.00 mg L^− 1^ IBA) and T6 (4.00 mg L^− 1^ IBA), respectively, while the lowest average number of roots per plantlet (1.16) was recorded with the control treatment T1 (0.00 mg L^− 1^ IBA).

The data shown in Table 3; Figs. 4, 5, 6, and 7 made it evident how the genotype rootstock behaved in relation to the average number of roots per plantlet as influenced by IBA. The Garnem genotype produced the highest significant number of roots (7.06), which was followed by the Nemared genotype (3.16), while the lowest average root number per plantlet (2.79) was recorded with the Okinawa.

**Table 3:** The effect of varying IBA concentrations on the average number of roots per plantlet of the Okinawa, Garnem, and Nemared *Peach* rootstocks.

Treatment (B)	Rootstock (A)			
	Okinawa	Nemared	Garnem	Average (B)
T1 (0.00 mg L^− 1^ IBA)	0.00 g	0.00 g	3.50 c–g	1.16 D
T2 (0.5 mg L^− 1^ IBA)	1.50 e–g	1.00 f–g	5.33 b–e	2.61 CD
T3 (1.00 mg L^− 1^ IBA)	2.91 d–g	2.00 d–g	6.11 b–d	3.67 BC
T4 (2.00 mg L^− 1^ IBA)	4.33 b–f	5.00 b–f	7.33 b–c	5.55 AB
T5 (3.00 mg L^− 1^ IBA)	5.00 b–f	8.00 b	7.75 b	6.91 A
T6 (4.00 mg L^− 1^ IBA)	3.00 d–g	3.00 d–g	12.33 a	6.11 A
Average (A)	2.79 B	3.16 B	7.06 A	

*The LSD test shows that the means of each factor and their interaction, denoted by the same letters, are not significantly different from one another at *P* ≤ 0.05.

Regarding the interaction between IBA and genotypes as shown in Table 3; Figs. 4, 5, 6, and 7 the rootstocks showed significantly different responses to the root number per plantlet with relation to the IBA concentrations tested; the maximum significant value (12.33 as an average number of roots per plantlet) was recorded in T6 (4.00 IBA mg L^− 1^) with the Garnem genotype, followed by the Nemared genotype, which recorded 8.00 in T5 (3.00 IBA mg L^− 1^), and the Okinawa genotype, which recorded 5.00 in T5 (3.00 IBA mg L^− 1^). While T1 (with the absence of IBA), root formation did not significantly occur with the Okinawa and Nemared genotypes under this study. Whereas the genotype of Garnem was rooted 3.50 (as an average number of roots per plantlet) without the addition of IBA, which could be due to the presence of sufficient internal auxin for the rooting process to occur^39^.

According to our findings, the rootstock Garnem had the greatest root number per plantlet (Fig. 4) and in vitro rooting rate. For the rootstocks Garnem, Okinawa, and Nemared, the corresponding rooting rates were 100%, 83.33%, and 75.00% at a level of 2.0 mg L^− 1^ of IBA. We found that the interaction between genotype and IBA concentration had a significant effect, which supports the need to optimize IBA concentration for each genotype.

The results observed concerning the percentage of rooting, root length, and the number of roots per plantlet are similar to those found in the literature for the different species of the genus *Prunus*, which found that root formation depended on the concentration of auxins used^10,14,32,37,49–52^. Whereas^10^ found that the rootstock Capdeboscq had the greatest root number per shoot and in vitro rooting rate. At 1.0 mg L^− 1^ of IBA, the corresponding rooting rates for the rootstocks Capdeboscq, GF677, and VP411 were 100%, 64%, and 64%, respectively. For the VP417 selection, rooting rates of 64.0% were linked to an IBA level of 2.0 mg L^− 1^. In response to the level of 2.0 mg L^− 1^ of IBA, the maximum number of roots per shoot for the rootstocks Capdeboscq and GF677 was 9.6 and 5.2, respectively. The selections VP411 and VP417 responded to the level of 1.0 mg L^− 1^ of IBA with the highest root numbers (3.6 and 3.9, respectively). When 0.5 mg L^− 1^ of IBA was used in Peach cerasifera, 4.4 roots were formed per plantlet, yielding a 97% in vitro rooting rate^53^.

**Fig. 8:**
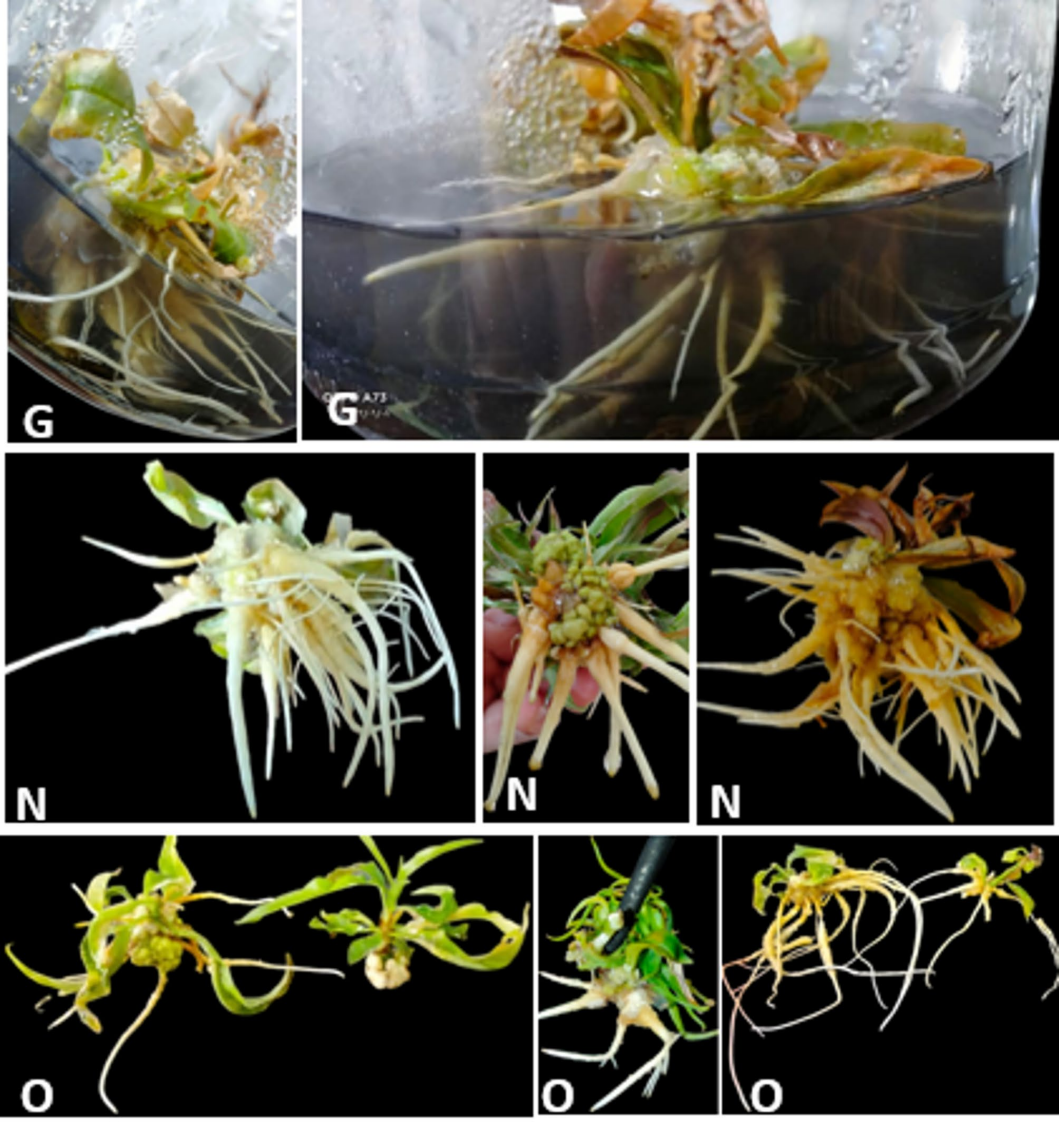
Adverse root development and callus formation at the base of the “Garnem” (G), “Okinawa” (O), and “Nemard” (N) plantlets in vitro as a result of high IBA concentration (4 mg L^− 1^).

Despite the number of roots, this is a significant factor for increased percentages of plant survival during acclimatization^37^. Thus, in order to promote rhizogenesis^14^ and the formation of many roots^16^, exogenous auxins are needed in the early stages^16^. It is important to adjust the IBA level in the medium for each genotype of *peach* rootstocks to induce and develop normal roots per plantlet. However, the current study demonstrated that callus and abnormal roots formed when the concentration of IBA was increased to 4.00 mg L^− 1^ with the three rootstocks (Fig. 8). These findings are consistent with those of^54^, who found that peach rootstock GF 677’s normal root development was inhibited and callus was induced by higher levels of IBA (4.0 mg L^− 1^).

### Acclimatization stage

**Fig. 9:**
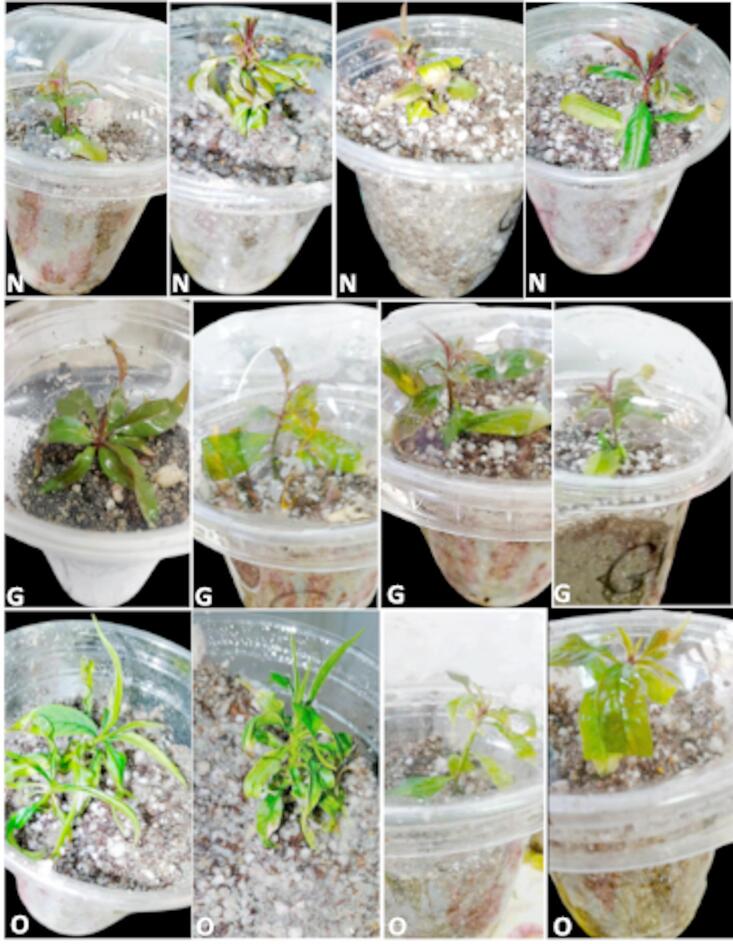
Acclimatization of the (N) Nemared, (G) Garnem and (O) Okinawa peach rootstocks.

The acclimatization phase is a very difficult step affected by different factors such as genotype, medium composition, and plant growth regulators^55,56^. Moreover^57^, reported that difficult-to-root plants often perform poorly during acclimatization, and in vitro rooting can increase the survival and quality of plants. Therefore, the acclimatization stage is one of the important stages in tissue culture. The average survival percentages were 90, 75, and 93% for the three peach rootstocks of the Okinawa, Nemared, and Garnem, respectively (Fig. 9).

This outcome is consistent with that of^52^, who demonstrated that within the GF677 rootstock, rooted plantlets were successfully acclimated and moved to a potting mix with 90% survival. They then grew naturally after being strengthened and moved to a soil mixture consisting of perlite, sand, and soil in a ratio of 1:2:1. Furthermore^45^, showed that 90% of the plantlets survived during the acclimatization phase in the greenhouse when working with GF677 hybrid rootstock and Rabi cultivar. According to^46^, when plantlets were placed in soil, the ex-vitro survival rate for Garnem peach rootstock was 95%. Also^34^, reported that the survival rates for the Cadaman and Garmen rootstocks were 83% and 63.3%, respectively, following acclimatization.

## Conclusion

The present study successfully micropropagated the Okinawa, Nemared, and Garnem peach rootstocks in vitro, with promising results for large-scale propagation. The IBA treatments resulted in a significant difference in the root number per shoot, root length, and in vitro rooting rate of the three rootstocks, with the Garnem genotype exhibiting the highest root number per shoot, root length, and in vitro rooting rate. The use of 2.0 mg L^− 1^ of IBA produced high rooting rates (100%, 83.33%, and 75%) with the Garnem, Okinawa, and Nemared peach rootstocks, respectively, which improved the average survival percentages (93%, 90%, and 75%) for the three rootstocks following acclimatization. Additionally, the current study showed that when the IBA concentration was raised to 4.00 mg L^− 1^ with the three rootstocks, callus and abnormal roots developed.

## Data Availability

The authors affirm that the article contains all pertinent information. Data from this study can be requested by contacting Galal Eliwa at geliwa2002@du.edu.eg.
